# Urinary and Fecal Metabonomics Study of the Protective Effect of Chaihu-Shu-Gan-San on Antibiotic-Induced Gut Microbiota Dysbiosis in Rats

**DOI:** 10.1038/srep46551

**Published:** 2017-04-20

**Authors:** Meng Yu, Hong-Mei Jia, Chao Zhou, Yong Yang, Li-Li Sun, Zhong-Mei Zou

**Affiliations:** 1 Institute of Medicinal Plant Development, Chinese Academy of Medical Sciences and Peking Union Medical College, Beijing 100193, P. R. China

## Abstract

Accumulating evidence suggests that the gut microbiota dysbiosis and their host metabolic phenotype alteration is an important factor in human disease development. A traditional Chinese herbal formula, Chaihu-Shu-Gan-San (CSGS), has been effectively used in the treatment of various gastrointestinal (GI) disorders. The present study was carried out to investigate whether CSGS modulates the host metabolic phenotype under the condition of gut microbiota dysbiosis. The metabonomics studies of biochemical changes in urine and feces of antibiotic-induced gut microbiota dysbiosis rats after treatment with CSGS were performed using UPLC-Q-TOF/MS. Partial least squares-discriminate analysis (PLS-DA) indicated that the CSGS treatment reduced the metabolic phenotype perturbation induced by antibiotic. In addition, there was a strong correlation between gut microbiota genera and urinary and fecal metabolites. Moreover, the correlation analysis and the metabolic pathway analysis (MetPA) identified that three key metabolic pathways including glycine, serine and threonine metabolism, nicotinate and nicotinamide metabolism, and bile acid metabolism were the most relevant pathways involved in antibiotic-induced gut microbiota dysbiosis. These findings provided a comprehensive understanding of the protective effects of CSGS on the host metabolic phenotype of the gut microbiota dysbiosis rats, and further as a new source for drug leads in gut microbiota-targeted disease management.

The gut microbiota, the population of microorganisms that inhabit the GI tract, are now recognized as a significant factor in determining host health and with respect to conferring an extended metabolic capacity on the host. Gut microbiota colonization can be influenced by alterations in diet, age, antibiotic use and other environmental factors^1^,^2^,^3^. In turn, gut microbiota dysbiosis is known to influence host metabolic phenotype and also act as a causative factor in many forms of human diseases such as obesity, type 2 diabetes, and cardiovascular disease^3^,^4^. Hence, evaluation of the host-gut microbiota co-metabolism interactions may provide new insight into the important role of gut microbiota on the host health.

Germ-free models provide valuable information on microbial-mammalian co-metabolism^5^,^6^. However, the gut of these models are underdeveloped, do not provide a true picture of ‘normal’ mammalian physiology and are as such not an optimal model for characterizing microbiota-mammalian metabolic interaction in ‘normal’ animals^7^. Therefore, an alternative approach is to employ conventional animals with normal gut physiology, treated with antimicrobials and very closely mimics the actual environment of the host gut and its commensals. Furthermore, information relating to the interactions between the host genome and gut microbiota is usually captured in the metabolic signature. Metabonomics strategies, therefore, provide a window into the host metabolic phenotype and permit the influence of the gut microbiota and the interactions on the host co-metabolism to be studied.

It has been fully evidenced that the gut microbiota dysbiosis along with the metabolic phenotype alteration was associated with various diseases, such as obesity, diabetes and atherosclerosis. Furthermore, evidence also establishes that both the pathological symptoms and the gut microbiota dysbiosis can be alleviated by traditional Chinese medicines (TCMs)^8^,^9^,^10^. The above mentioned facts encouraged us to address the hypothesis that TCM decoctions potentially work directly (as prebiotics) and/or indirectly (under certain pathological conditions) to induce favorable changes in the gut microbiota and further improved microbial-host co-metabolism. CSGS is a TCM decoction composed of seven commonly used Chinese herbs: viz., the root of *Bupleurum chinense* DC. (Chai-Hu), the pericarps of *Citrus reticulata* Blanco (Chen-Pi), the root of *Paeonia lactiflora* Pall. (Bai-Shao), the fruit of *Citrus aurantium* L. (Zhi-Qiao), the root of *Cyperus rotundus* L. (Xiang-Fu), the root of *Ligusticum chuanxiong* Hort. (Chuan-Xiong) and the root of *Glycyrrhiza uralensis* Fisch. (Gan-Cao). The chemical constituents in CSGS formulation were qualitatively and quantitatively investigated by an optimized LC–LTQ-Orbitrap method^11^. It has been used in China for the clinical treatment of various GI disorders including gastric ulcers and inflammation related to helicobacter pylori infection, GI infections or diarrhea, chronic erosive gastritis, and depression^12^. The metabonomics studies suggest that antidepressant effect of CSGS could mediate the disturbance of multiple metabolic pathways^13^,^14^. Emerging evidence from patients with depressive disorders demonstrated that gut microbiota dysbiosis associated with the etiology and biological mechanisms of depression^15^,^16^. These observations highlight that the association of the antidepressant effects of CSGS might potentially work directly to regulate the dysbiosis of gut microbiota and abnormal host metabolic phenotype. However, the current knowledge is inadequate in understanding the regulatory mechanism of CSGS to the gut microbiota dysbiosis, which will become a focal point to evaluate the pharmacodynamics and mechanisms of TCM formulas.

In the present study, we employ an antibiotic-based model to directly probe the dynamic effect of the microbiotal contribution to urinary and fecal metabolome composition. In turn, a combined 16S rRNA gene sequencing and UPLC-Q-TOF/MS-based metabonomics method was established to provide a comprehensive understanding of the protective effects of CSGS on the host metabolic phenotype of the gut microbiota dysbiosis rats.

## Result and Discussion

### Gut microbiota dysbiosis of the antibiotic treatment

**Figure 1:**
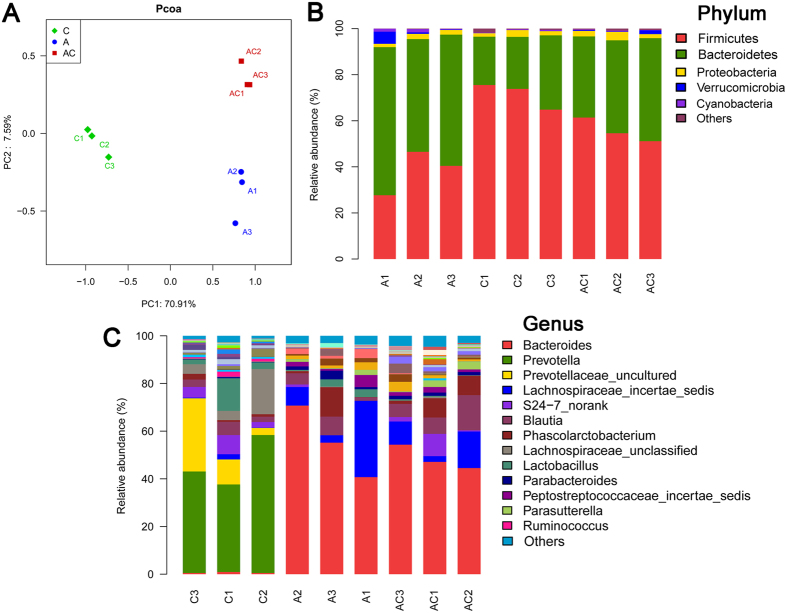
The gut microbiota patterns of control group (C), antibiotic group (A) and CSGS + antibiotic group (AC) differentiated by PCoA (**A**). The gut microbiota composition profiles at the phylum (**B**) and genus (**C**) levels in the control and model group revealed by 16S rRNA gene sequencing (each color represents one bacterial phylum or genus).

Fecal microbiota composition profiles were analyzed by 16S rRNA gene sequencing-based method. To compare community patterns, a principal coordinate analysis (PCoA) was used. A scatter plot based on PCoA scores from the sequences at OUT level with >97% similarity showed a clear separation of the community composition between the antibiotic group and control group (Fig. 1A). Moreover, around 99% of the total bacterial abundance was classified into five phyla, while the rest was allocated to various unclassified bacteria. The dominant phyla including *Firmicutes* and *Bacteroidetes* and the antibiotic treatment resulted in significant reduction in levels of *Firmicutes* and increase levels of *Bacteroidetes*, whereas these two disturbed gut microbiota phyla could be regulated after CSGS treatment (Fig. 1B). In addition, we also found 16 statistically significant differences between model and control groups at the genus level (Fig. 1C). In order to assure the accuracy and repeatability of results, we repeated the animal experiment (n = 6 per group) and also randomly chose three fecal samples from each group for second 16S rRNA sequencing, and the two experiment results are consistent (Figure S3). These results suggest that the antibiotic treatment led to significant changes of the gut microbiota at the phylum and genus level in the antibiotic group compared with the control group.

### Effect of CSGS treatment on the body weight in antibiotic-treated rats

**Figure 2:**
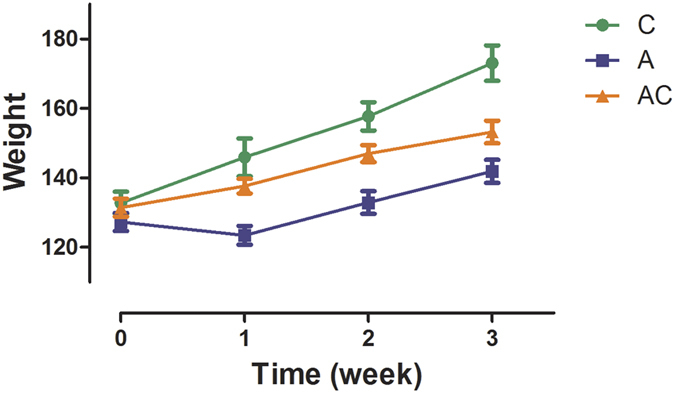
Increasing of the body weight in rats (C: control group, A: antibiotic group, AC: CSGS + antibiotic group).

A significant reduction in body weight in the antibiotic group was observed from 1 to 3 weeks post-treatment compared with the control group. CSGS showed a significant increase in body weight compared to the antibiotic group (Fig. 2).

### Effect of CSGS treatment on urinary metabolic profiles of gut microbiota dysbiosis

**Figure 3:**
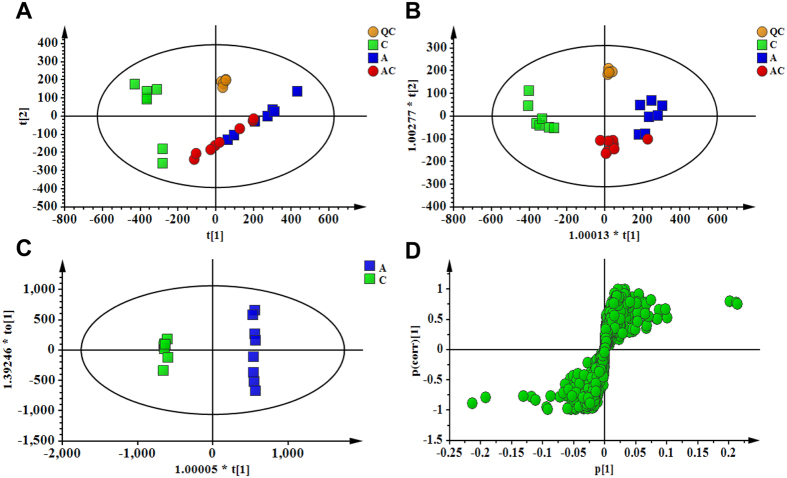
PCA score plot of urine samples collected from different treatment groups of rat in (**A**, *R*^*2*^X = 0.902, *Q*^*2*^ (cum) = 0.775) positive ion mode; PLS-DA score plot of urine samples collected from different treatment groups of rat in (**B**, *R*^*2*^X = 0.942, *R*^*2*^Y = 0.797, *Q*^*2*^ (cum) = 0.670) positive ion mode; OPLS-DA score plot and *S*-Plot of the control and antibiotic group in (**C**,**D**) positive ion mode. (**C**) Control group, (**A**) antibiotic group, (AC) CSGS + antibiotic group, (QC) quality control samples.

Metabolic profiles of urine samples in each group were analyzed by UPLC-Q-TOF/MS in positive ion mode. The typical base peak intensity (BPI) chromatograms of all experimental groups are shown in Figure S1. All the tested groups were discriminated in the principal component analysis (PCA) model (Fig. 3A). In order to provide better visualization for discriminating groups of samples from PCA and carrying out the class separating information of variables, supervised approach was performed. In this study, PLS-DA was used to evaluate the metabolic patterns of antibiotic-induced gut microbiota dysbiosis rats with and without CSGS treatment. The PLS-DA (Fig. 3B) analysis indicated that the metabolic profile of rats in the antibiotic group deviated from the control, which suggests that the antibiotic treatment induced significant biochemical changes. However, the metabolic profile of rats in CSGS treated group fairly differed from the antibiotic group but was similar to the control, indicating that the antibiotic-induced deviations significantly improved after CSGS treatment.

### Identification of potential urinary biomarkers associated with gut microbiota dysbiosis

**Figure 4:**
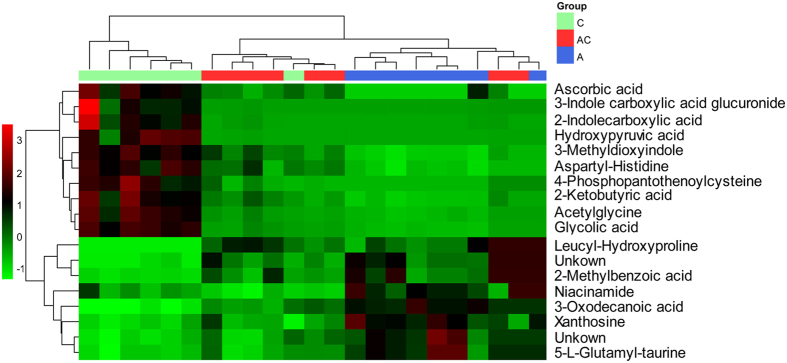
Significant changes in urinary metabolites are expressed as a heatmap showing metabolite changes in all treatment groups, detected by UPLC-Q-TOF/MS. (C) Control group, (A) antibiotic group, (AC) CSGS + antibiotic group.

The orthogonal to partial least squares-discriminate analysis (OPLS-DA) method was employed to sharpen an already established separation between the antibiotic group and control group in PLS-DA (Fig. 3C). The *S*-plot and VIP were used to select potential biomarkers (Fig. 3D). Ions far from the origin and VIP >1 could be considered as potential biomarkers responsible for the metabolic profile of gut microbiota dysbiosis. Results showed that 18 ions contributed to the clustering, their retention time, *m/z* and VIP values are listed in Table S1. Identification of the metabolites was performed based on the accurate mass and the collected MS^E^ spectra measurements via Q-TOF/MS and comparison of the data with literature and/or database resources. Databases, such as HMDB, METLIN, MassBank and KEGG were used for confirmation. They are leucyl-hydroxyproline (**U1**), 3-oxodecanoic acid (**U2**), glycolic acid (**U3**), hydroxypyruvic acid (**U4**), acetylglycine (**U5**), aspartyl-Histidine (**U6**), 4-phosphopantothenoylcysteine (**U7**), 2-methylbenzoic acid (**U8**), 3-indole carboxylic acid glucuronide (**U9**), 2-indolecarboxylic acid (**U10**), 3-methyldioxyindole (**U11**), niacinamide (**U12**), 5-L-glutamyl-taurine (**U13**), xanthosine (**U15**), 2-ketobutyric acid (**U17**) and ascorbic acid (**U18**). The varied tendencies of the identified urinary biomarkers related to gut microbiota dysbiosis are depicted in the heatmap for each treatment group (Fig. 4).

### Fecal metabolic profiles of gut microbiota dysbiosis with CSGS treatment

**Figure 5:**
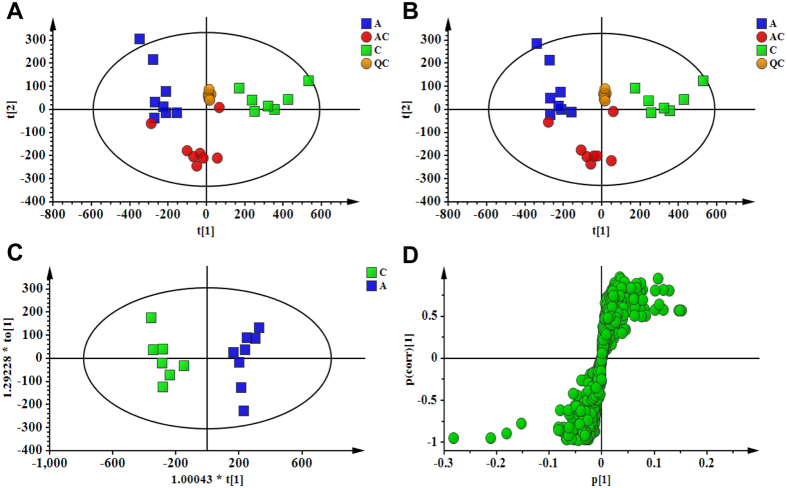
PCA score plot of feces samples collected from different treatment groups of rat in (**A**, *R*^*2*^X = 0.847, *Q*^*2*^ (cum) = 0.681) positive ion mode; PLS-DA score plot of feces samples collected from different treatment groups of rat in (**B**, *R*^*2*^X = 0.808, *R*^*2*^Y = 0.670, *Q*^*2*^ (cum) = 0.517) positive ion mode; OPLS-DA score plot and *S*-Plot of the control and antibiotic group in (**C**,**D**) positive ion mode. (**C**) Control group, (**A**) antibiotic group, (AC) CSGS + antibiotic group, (QC) quality control samples.

The metabolic profiles of feces samples from antibiotic-induced gut microbiota dysbiosis rats with and without CSGS treatment were characterized using UPLC-Q-TOF/MS in positive ion scan mode. The typical BPI chromatograms of all experimental groups are shown in Figure S2. The PCA and PLS-DA model were performed to evaluate the metabolic patterns of antibiotic-induced gut microbiota dysbiosis rats after CSGS treatment (Fig. 5A and B). The metabolic profile of rats in CSGS treated group fairly differed from the antibiotic group but was similar to the control group, indicating the deviations induced by antibiotic were significantly improved after CSGS treatment.

### Identification of potential fecal biomarkers associated with gut microbiota dysbiosis

**Figure 6:**
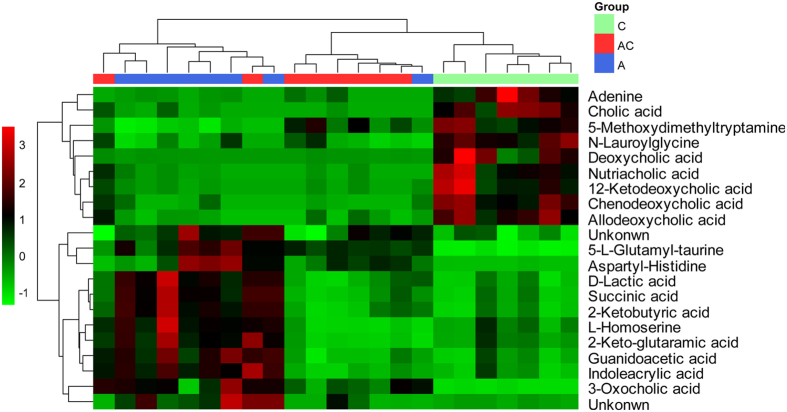
Significant changes in fecal metabolites are expressed as a heatmap showing metabolite changes in all treatment groups, detected by UPLC-Q-TOF/MS. (**C**) control group, (**A**) antibiotic group, (**AC**) CSGS + antibiotic group.

As shown in Fig. 5C and D, the OPLS-DA and *S*-Plot based on fecal metabolic profiles between the control group and the antibiotic group indicated 21 ions that contributed to the clustering in positive ion mode. Their VIP values are listed in Table S2. The metabolites were identified by means of accurate mass measurements via QTOF-MS and MS/MS product ion analysis and comparison of the data with literature and/or database resources. The analysis identified 21 metabolites in feces samples as potential biomarkers involved in gut microbiota dysbiosis. The metabolites are adenine (**F1**), D-lactic acid (**F2**), succinic acid (**F3**), 5-methoxydimethyltryptamine (**F4**), L-homoserine (**F5**), 2-ketobutyric acid (**F6**), 2-keto-glutaramic acid (**F7**), guanidoacetic acid (**F8**), indoleacrylic acid (**F9**), N-lauroylglycine (**F10**), 5-L-glutamyl-taurine (**F12**), aspartyl-histidine (**F14**), cholic acid (**F15**), 3-oxocholic acid (**F16**), deoxycholic acid (**F17**), chenodeoxycholic acid (**F18**), nutriacholic acid (**F19**), 12-ketodeoxycholic acid (**F20**) and allodeoxycholic acid (**F21**). The varied tendencies of the identified fecal biomarkers related to gut microbiota dysbiosis are depicted in the heatmap for each treatment group (Fig. 6).

### Statistical correlations between altered gut microbiota, and urinary and fecal metabolites associations with gut microbiota dysbiosis

**Figure 7:**
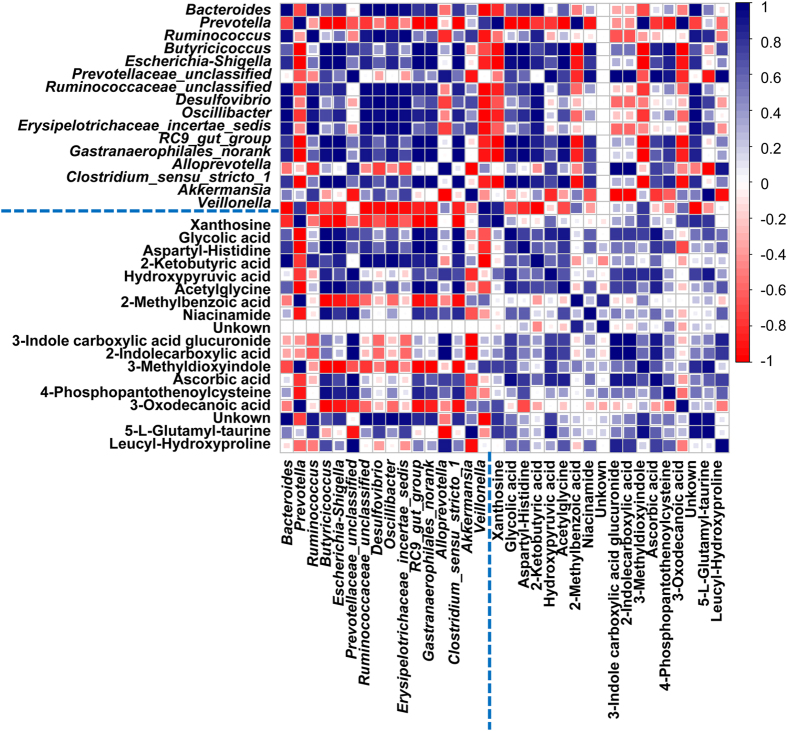
A correlation heatmap is used to represent significant statistical correlation values (*r*) between perturbed gut microbiota genera and altered urinary metabolites in antibiotic group and control group. Blue squares indicate significant positive correlations (*r* > 0.5, *p* < 0.05), white squares indicate nonsignificant correlations (*p* > 0.05), and red squares indicate significant negative correlations (*r* < −0.5, *p* < 0.05).

**Figure 8:**
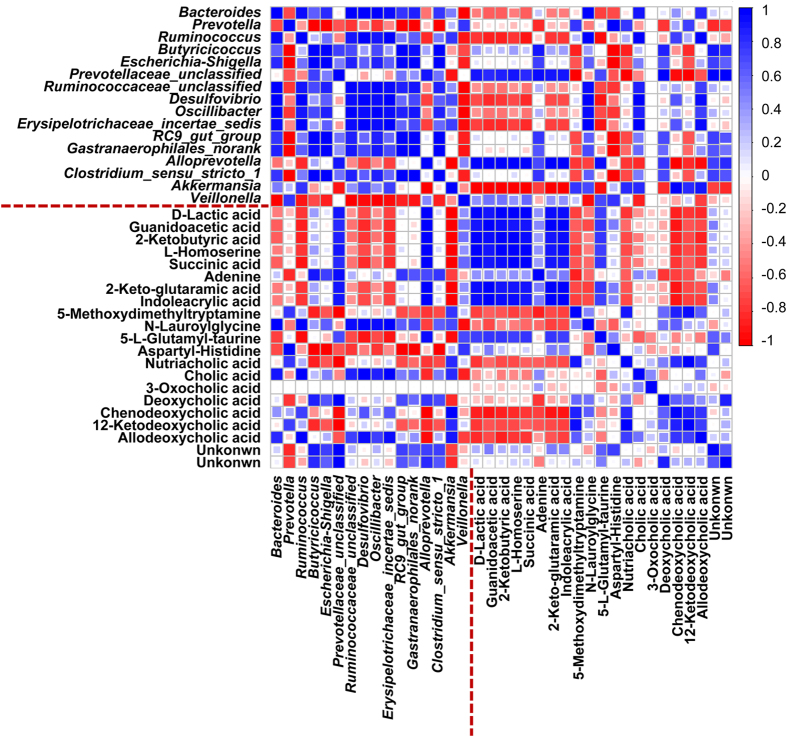
A correlation heatmap is used to represent significant statistical correlation values (*r*) between perturbed gut microbiota genera and altered fecal metabolites in antibiotic group and control group. Blue squares indicate significant positive correlations (*r* > 0.5, *p* < 0.05), white squares indicate nonsignificant correlations (*p* > 0.05), and red squares indicate significant negative correlations (*r* < −0.5, *p* < 0.05).

Pearson correlation analysis was used to identify potential links between altered gut microbiota genera and associations with urinary and fecal metabolites (r > 0.5 or r < −0.5, *p* < 0.05). Our correlation analysis identified multiple significant associations between the perturbed gut microbiota and altered urinary and fecal metabolites. As shown in Figs 7 and 8, hydroxypyruvic acid (**U4**) was positively related to *Ruminococcaceae_unclassified* and *Oscillibacter* but was negatively linked to *Veillonella*. Niacinamide (**U12**) displayed strong positive correlations with *Prevotella*. 2-ketobutyric acid (**U17**) and deoxycholic acid (**F17**) were determined to correlate positively with *Akkermansia*, while negative correlations with *Alloprevotella*. Guanidoacetic acid (**F8**) was found to be positively related to *Alloprevotella* and negatively related to *Akkermansia*. Significant positive correlations were observed between N-lauroylglycine (**F10**) and *Desulfovibrio* and *Erysipelotrichaceae_incertae_sedis*. These metabolites were involved in 3 key metabolic pathways including glycine, serine and threonine metabolism (**U4**, **U17**, **F8,** and **F10**), nicotinate and nicotinamide metabolism (**U12**), and bile acid metabolism (**F17**), and also achieved a complete metabolome contributing to the formation of the gut microbiota dysbiosis. In summary, the antibiotic treatment induced a significant taxonomic perturbation in the gut microbiota, which in turn substantially alters the metabolic phenotype of the gut microbiota, as evidenced by changes in diverse gut microbiota-related metabolites and metabolic pathways.

### Key metabolic pathways associations with gut microbiota dysbiosis validation

**Figure 9:**
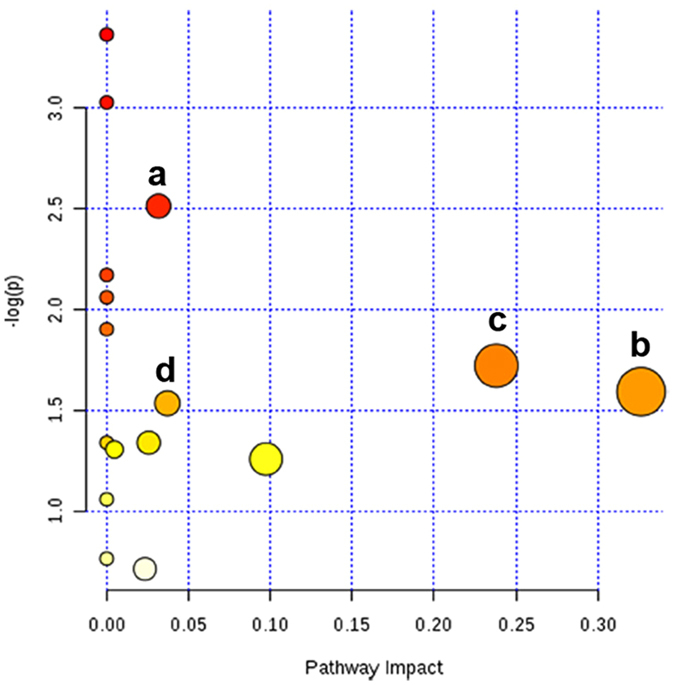
Summary of pathway analysis with MetPA. (a) Glycine, serine and threonine metabolism; (b) pantothenate and CoA biosynthesis; (c) nicotinate and nicotinamide metabolism; (d) bile acid metabolism.

To further validate these key metabolic pathways identified by statistical correlations, were the most relevant pathways to gut microbiota dysbiosis, a comprehensive metabolic network was mapped by means of MetaboAnalyst 3.0 (http://www.metaboanalyst.ca/) by integration of all potential biomarkers identified in present research^17^. The impact value with the MetPA was applied to evaluate the importance of the pathways on the development of gut microbiota dysbiosis (Fig. 9 and Table S3). As a result, four disturbed metabolic pathways were considered as the most relevant pathways involved in antibiotic-induced gut microbiota dysbiosis (impact > 0.01)^18^. They are glycine, serine and threonine metabolism, pantothenate and CoA biosynthesis, nicotinate and nicotinamide metabolism and bile acid metabolism. Among them, three metabolic pathways were identified both by statistical correlations analysis and by MetPA. Therefore, glycine, serine and threonine metabolism, nicotinate and nicotinamide metabolism, and bile acid metabolism were recognized as the key metabolic pathways in the formation of antibiotic-induced gut microbiota dysbiosis.

### Glycine, serine and threonine metabolism

The pathway of glycine, serine and threonine metabolism supplies important energy metabolism precursors to enter into the citrate cycle^19^. The results showed a decrease in levels of metabolites including hydroxypyruvic acid (**U4**) and acetylglycine (**U5**) in urine samples of gut microbiota dysbiosis rats, suggesting the deficiency of energy metabolism in the host of gut microbiota dysbiosis. Meanwhile, we observed up-regulated feces levels of L-homoserine (**F5**) and guanidoacetic acid (**F8**), which may have played a role in disturbing gut microbiota homeostasis. In addition, 2-ketobutyric acid (**U17** or **F6**), a gut microbiota co-metabolite^20^, was measured both in urine and feces. The levels of these metabolites decreased in urine but increased in feces. Taken together, the decreased urine levels of hydroxypyruvic acid (**U4**), acetylglycine (**U5**) and 2-ketobutyric acid (**U17**) and increased feces levels of L-homoserine (**F5**), guanidoacetic acid (**F8**) and 2-ketobutyric acid (**F6**) indicated the dysbiosis of gut microbiota. The CSGS treatment significantly reversed these abnormal metabolites, suggesting CSGS could effectively ameliorate the abnormal change of glycine, serine and threonine metabolism.

### Nicotinate and nicotinamide metabolism

Niacinamide (**U12**) is involved in the nicotinamide adenine dinucleotide (NAD) salvage pathway in mammals, a process which requires adenosine triphosphate (ATP)^21^. ATP depletion affects NAD regeneration resulting in the accumulation of niacinamide (**U12**)^22^. Here, increase in the urinary metabolites of niacinamide (**U12**) may be due to ATP depletion which is subsequently excreted in urine. The pretreatment with CSGS has regulatory effect on nicotinate and nicotinamide metabolism perturbation.

### Bile acid metabolism

Bile acids are known to exert several biological effects *in vivo*, such as the role of ‘signaling molecules’ to regulate metabolic homeostasis by activating diverse nuclear receptors^23^. Meanwhile, specific microbial bile acid co-metabolites present in metabolic pathway changes regulated by farnesoid X receptor indicate a broad signaling role for bile acids and highlight the symbiotic microbial influences in bile acid homeostasis in the host^24^. As one example, deoxycholic acid (**F17**) is one of the major secondary bile acids derived through dehydroxylation of bacteria in the gut. In this study, decreased levels of cholic acid (**F15**), deoxycholic acid (**F17**), chenodeoxycholic acid (**F18**), nutriacholic acid (**F19**) and allodeoxycholic acid (**F21**) were observed in feces samples of gut microbiota dysbiosis rats, which can be explained bile acid metabolism involved in the dysbiosis of gut microbiota. After CSGS treatment, all the derivations of bile acid metabolites were corrected, indicating the regulation of the perturbation of bile acid metabolism contributes to the gut microbiota dysbiosis effect of CSGS.

Other microbe-derived metabolites and metabolic pathways altered in antibiotic-induced gut microbiota dysbiosis rats in their urine and feces, and these may have potential as biomarkers of gut microbiota dysbiosis. Although our study was not designed to unravel the complex mechanisms linking gut microbiota dysbiosis with the detected metabolic alterations, it is possible that a reduction in several genera known to have enzymatic activity on the metabolic phenotype of the host may induce a change in their urine and feces excretion. Future work should aim to explore the role of these metabolites in gut microbiota dysbiosis pathogenesis and to determine their biomarker potential in large human cohorts.

Taken together, the key metabolic pathways were identified both statistical correlations analysis and MetPA, play important roles in the host metabolic phenotype of gut microbiota dysbiosis. Concerning the integral nature of a specific gut microbiome and metabolome, gut microbiota dysbiosis could result in significant alterations in the extracellular metabolic phenotype of the host, which may account for important findings commonly encountered in pathology, toxicology or drug metabolism studies^25^. The current model of antibiotic-induced gut microbiota dysbiosis is explained largely by 16S rRNA gene sequencing analysis and the urinary and fecal metabolic phenotype. TCMs have been developed and advocated for use in the treatment of many diseases for over 2500 years in China, which can modulate gut microbiota structure, thereby as a new source of drugs for the treatment of obesity, diabetes, and atherosclerosis^8^,^9^,^10^. Our results suggest that the antibiotic-induced disturbed metabolic phenotype of the host can be modulated by the dietary intervention of CSGS extract. In particular, this treatment enriched the number of beneficial bacteria, such as *Lactobacillus* spp. in the gut, and directly contributed to the improvement of the host metabolic phenotype homeostasis.

## Conclusions

A combined 16S rRNA gene sequencing and urinary and fecal metabonomics method have been established to evaluate the protective effects of CSGS on the metabolic phenotype of the host on the antibiotic-induced gut microbiota dysbiosis rats. Pattern recognition with multivariate statistical analysis allowed the metabolic profiles of urine and feces of antibiotic-induced gut microbiota dysbiosis group clearly separated from the control group, and the CSGS treated group was closer to the control group. In addition, correlation analysis and MetPA identified three key metabolic pathways including glycine, serine and threonine metabolism, nicotinate and nicotinamide metabolism, and bile acid metabolism were the most relevant pathways involved in antibiotic-induced gut microbiota dysbiosis. Taken together, these results indicate that TCM formulas may be used as prebiotics to modulate gut microbiota structure and metabolic phenotype of the host, and further as a new source for drug leads in gut microbiota-targeted disease management.

## Methods

### Reagents and Materials

HPLC-grade acetonitrile was purchased from J.T. Baker (Phillipsburg, NJ, SA). Ultrapure water (18.2 MΩ) was prepared with a Milli-Q water purification system (Millipore, France). Formic acid (HPLC grade) was obtained from Tedia (Fairfield, USA). Leucine-enkephalin and ammonium formate were purchased from Sigma Aldrich (St. Louis, USA). Imipenem/cilastatin sodium (with equal quantities, trade named Tienam), a broad-spectrum β-lactam antibiotic was purchased from Hangzhou MSD Pharmaceutical Company Limited (Hangzhou, China). All other used chemicals were of analytical grade.

### Rats and treatments

Twenty-four healthy male Wistar rats (weighing 100 ± 10 g) were obtained from the Institute of Laboratory Animal Science, CAMS & PUMC (Beijing, China). The rats were housed individually in cages and maintained (20–25 °C and 40–60% humidity) under controlled conditions of 12 h light-12 h dark cycles (lights on from 6:00 a.m.–6:00 p.m.) and water available *ad libitum*.

The rats were randomly divided into three groups (n = 8): (1) Control group (C), orally normal saline; (2) Antibiotic group (A), dosed with antibiotic imipenem/cilastatin sodium at a daily dose of 50 mg/kg of body weight from day 1 to day 21^25^; (3) CSGS + antibiotic treated group (AC), rats received the same dose of antibiotic imipenem/cilastatin sodium for 21 days, and orally CSGS water extracts at the dose of 7.0 g kg^−1^ once daily for 21 days. The volume of water and chow consumed and body weight of each rat was recorded carefully every day.

### Sample Collection and Preparation

All rats in each group were housed in metabolic cages (1 per cage) for collecting the 24 h urine samples at day 20. A total of 23 urine samples (except one of control group) were collected and stored at −80 °C until analysis.

Feces samples were collected at day 20 of the each group. At least 2 pellets of feces were collected from each rat, transferred into sterile conical tubes, immediately frozen in liquid nitrogen and stored at −80 °C. A total of 23 feces samples (except one of control group) were collected and divided into two parts for microbiological (PCR-DGGE) and metabonomics analysis, respectively.

The study was approved by the Ethics Committee of the Institute of Medicinal Plant Development, CAMS & PUMC. All experimental procedures were performed in accordance with relevant guidelines and regulations approved by the Ethics Committee of the Institute of Medicinal Plant Development, CAMS & PUMC.

### 16S rRNA microbial community analysis

Feces samples DNA were extracted using the E.Z.N.A.^®^ Soil DNA Kit (Omega Bio-Tek, Norcross, GA, USA) according to the manufacturer’s protocols. The V3-V4 regions of the 16S rRNA gene was PCR-amplified in triplicate using custom barcoded universal bacterial primers (338F 5′-barcode-ACTCCTACGGGAGGCAGCA)-3′ and 806R 5′-GGACTACHVGGGTWTCTAAT-3′) with the following protocol: An initial DNA denaturation step at 95 °C for 3 min, 27 cycles of DNA denaturation at 95 °C (30 seconds), an annealing step at 55 °C (30 seconds), an elongation step at 72 °C (45 seconds), and a final elongation step at 72 °C (10 min). Triplicates were pooled, confirmed by electrophoresis on a 2% agarose gel, cleaned with the AxyPrep DNA kit (AXYGEN, Tewksbury, MA, USA), and sequenced on the Illumina HiSeq platform. 16S rRNA gene sequences were analyzed using the QIIME software package. All sequences were used for the comparison of the relative abundance of bacterial taxa. Representative sequences were clustered into Operational taxonomic units (OTUs) using Usearch^26^ and classified against the Greengenes Database^27^ according to 97% similarity. Any OTUs present less than five times among all samples were removed from the analysis. Statistical analysis of Bray-Curtis dissimilarities calculated using the relative abundance of bacterial genera was conducted using R (version 3.2.1) and the adonis function in the R package ‘vegan’.

### Urinary and fecal metabonomics

#### Sample Preparation

Urine samples were thawed at room temperature before analysis and centrifuged at 13,000 rpm at 4 °C for 10 min. The supernatant was diluted at a ratio of 1:1 with water and an aliquot of 5 *μ*L was injected for UPLC-Q-TOF/MS analysis.

Feces samples were first weighed and homogenized on ice. Fecal extracts were prepared by mixing 100 mg of feces samples with 500 *μ*L of ice cold water, vortexed and centrifuged (15 min, 13,000 rpm, 4 °C). The supernatant (fecal solution) were collected, and the remaining pellet was further extracted with 500 *μ*L of ice cold methanol. Supernatants obtained from two runs of extraction were combined and centrifuged at 13,000 rpm for 15 min at 4 °C and an aliquot of 5 *μ*L was injected for UPLC-Q-TOF/MS analysis.

#### UPLC-Q-TOF/MS analysis

Chromatographic analysis was performed on Waters ACQUITY UPLC System (Waters Corp. Milford, USA). The urine samples were performed on an Acquity UPLC HSS T3 column (100 mm × 2.1 mm, 1.8 *μ*m) and the feces samples were analyzed on an Acquity UPLC BEH C18 column (100 mm × 2.1 mm, 1.7 *μ*m). The columns were maintained at 40 °C and eluted at a flow rate of 0.45 mL/min. The mobile phase for urine samples was composed of water (A) and acetonitrile (B) each containing 0.1% formic acid. The gradient program for urine samples was optimized as follows: 0–0.5 min, 1% B; 0.5–7 min, 1% B to 10% B; 7–15 min, 10% B to 50% B; 15–17 min, 50% B to 100% B; 17–19 min, washing with 100% B, and 19–20 min, equilibration with 1% B. The mobile phase for feces samples was composed of solvents A (2 mM ammonium formate in 95% H_2_O/5% acetonitrile + 0.1% formic acid) and B (2 mM ammonium formate in 95% acetonitrile/5% H_2_O + 0.1% formic acid). The gradient program for feces samples was optimized as follows: 0–0.5 min, 1% B; 0.5–5 min, 1% B to 30% B; 5–13 min, 30% B to 50% B; 13–17 min, 50% B to 100% B; 17–19 min, washing with 100% B, and 19–20 min, equilibration with 1% B. The eluent from the column was directed to the mass spectrometer without split.

A Waters SYNAPT G2HDMS (Waters Corp., Manchester, UK) was used to carry out the mass spectrometry with an electrospray ionization (ESI) source operating in positive ion mode. The parameters were set as previously described^28^. Briefly, capillary voltage, 3.0 KV; sample and extraction cone voltage, 40 V and 4.0 V; desolvation gas rate and temperature, 800 L/h and 400 °C; cone gas rate, 40 L/h; source temperature, 100 °C; scan time and inter scan delay, 0.15 and 0.02 s. Leucine-enkephalin was used as the lockmass in all analyses ([M + H]^+^  = 556.2771) at a concentration of 0.5 *μ*g/mL with a flow rate of 5 *μ*L/min. Data was collected in centroid mode from *m/z* 50 to *m/z* 1500.

#### Method validation

To ensure the stability of sequence analysis, a quality control (QC) sample was prepared by pooling the same volume (10 *μ*L) from each urine and feces samples and then preparing the pooled QC sample in the same way as the samples. The pooled QC sample was analyzed randomly through the analytical batch to evaluate stability. Ten ions were extracted for method validation. The repeatability of the method was evaluated using 6 replicates by analyzing QC sample. The precision of the injection was assessed using 6 replicated analyses of the same urine and feces samples. The relative standard deviations (R.S.D%) of the retention time and *m/z* were shown in Table S4.

#### Data processing and multivariate analysis

The resulting MS data were first processed by the MarkerLynx Applications Manager version 4.1 (Waters Corp., Manchester, UK). This process included integration, normalization and alignment the intensities of peaks, and then give a list of *m/z* and retention time with corresponding intensities for each metabolites from every sample in the positive data set. The processed data list was then imported to SIMCA-P software package (v13.0, Umetric, Umea°, Sweden) for PCA and OPLS-DA. The PCA method was carried out to investigate whether each group can be separated and to find out their metabolic distinction. The OPLS-DA was used to pick out discriminating ions contributing to the classification among the experimental samples, and the results were visualized in the form of score plots to display the group clusters and *S*-plot to show variables contributing to the classification. In the OPLS-DA model, the variables responsible for differentiating antibiotic group and control group were selected as potential biomarkers of the diseases progression by the variable importance of project (VIP) value and the *S*-plot statistics.

### Statistical analysis

One-way ANOVA was performed using the Statistical Package for Social Science program (SPSS 16.0, Chicago, USA). The significance threshold was set at *P* < 0.05 for this test.

## Additional Information

**How to cite this article**: Yu, M. *et al*. Urinary and Fecal Metabonomics Study of the Protective Effect of Chaihu-Shu-Gan-San on Antibiotic-Induced Gut Microbiota Dysbiosis in Rats. *Sci. Rep.* **7**, 46551; doi: 10.1038/srep46551 (2017).

**Publisher's note:** Springer Nature remains neutral with regard to jurisdictional claims in published maps and institutional affiliations.

## Supplementary Material

Supplementary Information
