# Supplementary material for: Urinary and Fecal Metabonomics Study of the Protective Effect of Chaihu-Shu-Gan-San on Antibiotic-Induced Gut Microbiota Dysbiosis in Rats
Source: Sci Rep. 2017 Apr 20;7:46551. doi: 10.1038/srep46551 (PMC5397834; doi:10.1038/srep46551)
Supplement: Supplementary Information [file srep46551-s1.pdf]

Urinary and Fecal Metabonomics Study of the Protective Effect of Chaihu-Shu-Gan-San on Antibiotic-Induced Gut Microbiota Dysbiosis in Rats

Meng Yu, Hong-Mei Jia, Chao Zhou, Yong Yang, Li-Li Sun, Zhong-Mei Zou*

Institute of Medicinal Plant Development, Chinese Academy of Medical Sciences and Peking Union Medical College, Beijing 100193, P. R. China.

*Correspondence and requests for materials should be addressed to Zhong-Mei Zou (Email: zmzou@implad.ac.cn; Tel: +86-010-57833290; Fax: +86-010-57833290)

Supplementary legends

Figure S1: Typical UPLC-Q-TOF/MS base peak intensity (BPI) chromatograms of urine samples in positive ion mode. (**A**) control group, (**B**) antibiotic group, (**C**) CSGS + antibiotic group.

Figure S2: Typical UPLC-Q-TOF/MS base peak intensity (BPI) chromatograms of feces samples in positive ion mode. (**A**) control group, (**B**) antibiotic group, (**C**) CSGS + antibiotic group.

Figure S3: The gut microbiota patterns of control group (C), antibiotic group (A) and CSGS + antibiotic group (AC) differentiated by PCoA (A). The gut microbiota composition profiles of each experimental group at the phyla (B) and genera (C) levels in the control and model group revealed by 16S rRNA gene sequencing (each color represents one bacterial phylum or genus). (**The result of the repeated animal experiment**)

Table S1: The potential urine biomarkers of antibiotic induced gut microbiota dysbiosis rats detected by UPLC-Q-TOF/MS.

Table S2: The potential feces biomarkers of antibiotic induced gut microbiota dysbiosis rats detected by UPLC-Q-TOF/MS.

Table S3: Result from ingenuity analysis with MetPA.

Table S4: The reproducibility and precision of UPLC-Q-TOF/MS method validation under the positive ion mode using QC samples (urine samples: **A**; feces samples: **B**).

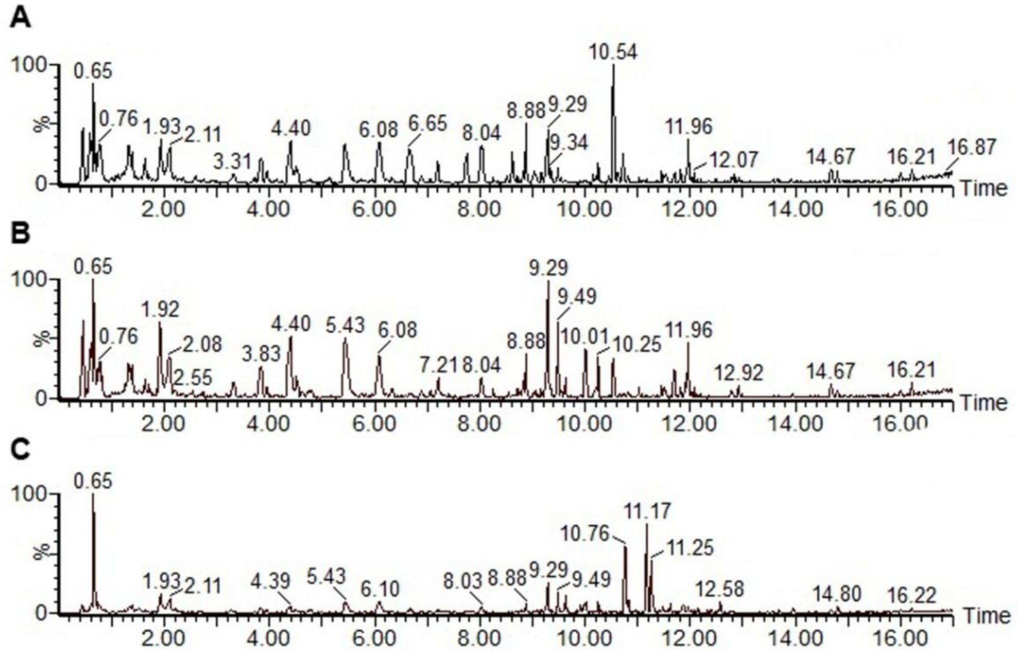

Figure S1. Typical UPLC-Q-TOF/MS base peak intensity (BPI) chromatograms of urine samples in positive ion mode. (**A**) control group, (**B**) antibiotic group, (**C**) CSGS + antibiotic group.

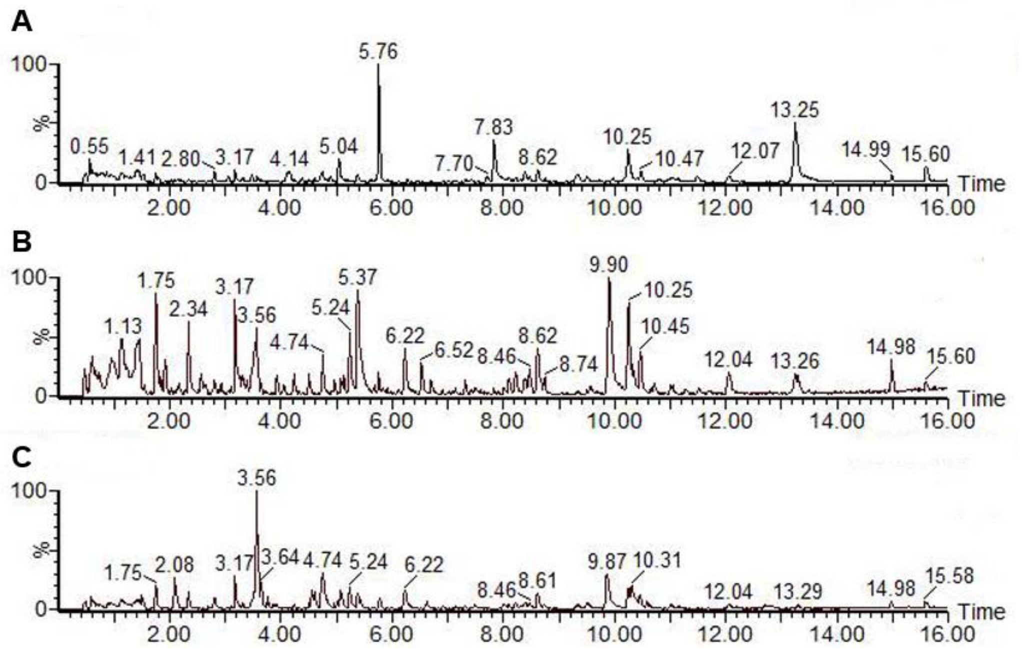

Figure S2. Typical UPLC-Q-TOF/MS base peak intensity (BPI) chromatograms of feces samples in positive ion mode. (**A**) control group, (**B**) antibiotic group, (**C**) CSGS + antibiotic group.

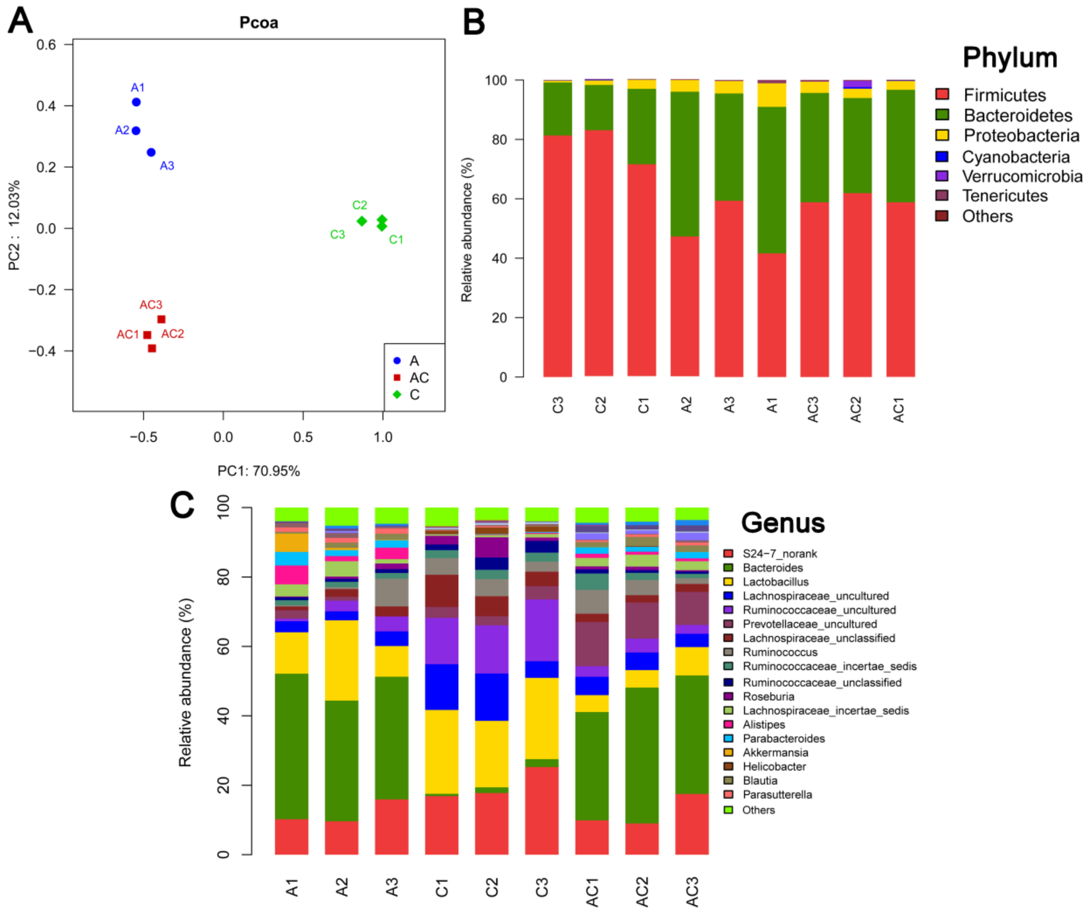

Figure S3: The gut microbiota patterns of control group (C), antibiotic group (A) and CSGS + antibiotic group (AC) differentiated by PCoA (A). The gut microbiota composition profiles of each experimental group at the phyla (B) and genera (C) levels in the control and model group revealed by 16S rRNA gene sequencing (each color represents one bacterial phylum or genus).

Table S1. The potential urine biomarkers of antibiotic induced gut microbiota dysbiosis rats detected by UPLC-Q-TOF/MS.

NO.	RT	m/z	VIP	metabolites	Adduct ion	Formula	Pathway
U1	1.87	267.1341	2.39	Leucyl-Hydroxyproline	$[M+Na]^+$	$C_{11}H_{20}N_2O_4$	Arginine and proline metabolism
U2	4.62	209.1259	3.39	3-Oxodecanoic acid	$[M+Na]^+$	$C_{10}H_{18}O_3$	—
U3	6.66	77.0387	5.85	Glycolic acid	$[M+H]^+$	$C_2H_4O_3$	Chlorocyclohexane and chlorobenzene degradation
U4	6.66	105.0336	3.71	Hydroxypyruvic acid	$[M+H]^+$	$C_3H_4O_4$	Glycine, serine and threonine metabolism
U5	6.66	118.0653	3.36	Acetylglycine	$[M+H]^+$	$C_4H_7NO_3$	Glycine, serine and threonine metabolism
U6	6.68	293.1134	3.41	Aspartyl-Histidine	$[M+Na]^+$	$C_{10}H_{14}N_4O_5$	Histidine metabolism
U7	6.68	425.0709	3.04	4-Phosphopantothenoylcysteine	$[M+Na]^+$	$C_{12}H_{23}N_2O_9PS$	Pantothenate and CoA biosynthesis
U8	7.41	137.0599	2.52	2-Methylbenzoic acid	$[M+H]^+$	$C_8H_8O_2$	Xylene degradation
U9	7.72	360.0688	2.54	3-Indole carboxylic acid glucuronide	$[M+Na]^+$	$C_{15}H_{15}NO_8$	Tryptophan metabolism
U10	7.73	162.055	7.36	2-Indolecarboxylic acid	$[M+H]^+$	$C_9H_7NO_2$	Tryptophan metabolism
U11	8.61	164.0707	12.01	3-Methyldioxyindole	$[M+H]^+$	$C_9H_9NO_2$	Tryptophan metabolism
U12	8.76	145.0288	3.16	Niacinamide	$[M+Na]^+$	$C_6H_6N_2O$	Nicotinate and nicotinamide metabolism
U13	9.29	255.0658	13.21	5-L-Glutamyl-taurine	$[M+H]^+$	$C_7H_{14}N_2O_6S$	Taurine and hypotaurine metabolism
U14	9.29	453.0795	4.07	Unkown	$[M+Na]^+$	—	—
U15	9.48	285.0758	12.59	Xanthosine	$[M+H]^+$	$C_{10}H_{12}N_4O_6$	Purine metabolism
U16	9.62	346.1056	2.79	Unkown	$[M+Na]^+$	—	—
U17	10.45	103.0543	2.82	2-Ketobutyric acid	$[M+H]^+$	$C_4H_6O_3$	Glycine, serine and threonine metabolism
U18	10.64	199.0211	3.24	Ascorbic acid	$[M+Na]^+$	$C_6H_8O_6$	Ascorbate and aldarate metabolism

Table S2. The potential feces biomarkers of antibiotic induced gut microbiota dysbiosis rats detected by UPLC-Q-TOF/MS.

NO.	RT	m/z	Metabolites	Adduct ion	Formula	VIP	Pathways
F1	0.76	136.061	Adenine	$[M+H]^+$	$C_5H_5N_5$	1.83	Purine metabolism
F2	1.15	91.0542	D-Lactic acid	$[M+H]^+$	$C_3H_6O_3$	1.45	Pyruvate metabolism
F3	1.16	119.0489	Succinic acid	$[M+H]^+$	$C_4H_6O_4$	1.42	Citrate cycle (TCA cycle)
F4	1.43	236.1852	5-Methoxydimethyltryptamine	$[M+NH_4]^+$	$C_{13}H_{18}N_2O$	1.99	—
F5	1.75	120.0808	L-Homoserine	$[M+H]^+$	$C_4H_9NO_3$	4.45	Glycine, serine and threonine metabolism
F6	1.75	103.0541	2-Ketobutyric acid	$[M+H]^+$	$C_4H_6O_3$	2.56	Glycine, serine and threonine metabolism
F7	2.34	146.0601	2-Keto-glutaramic acid	$[M+H]^+$	$C_5H_7NO_4$	4.14	Alanine, aspartate and glutamate metabolism
F8	2.34	118.0651	Guanidoacetic acid	$[M+H]^+$	$C_3H_7N_3O_2$	2.74	Glycine, serine and threonine metabolism
F9	2.34	188.0704	Indoleacrylic acid	$[M+H]^+$	$C_{11}H_9NO_2$	2.38	Styrene degradation
F10	3.59	258.2058	N-Lauroylglycine	$[M+H]^+$	$C_{14}H_{27}NO_3$	2.15	Glycine, serine and threonine metabolism
F11	5.08	465.1997	Unkonwn	$[M+Na]^+$	—	2.37	—
F12	5.23	255.0648	5-L-Glutamyl-taurine	$[M+H]^+$	$C_7H_{14}N_2O_6S$	3.69	Taurine and hypotaurine metabolism
F13	5.39	463.1835	Unkonwn	$[M+Na]^+$	—	2.64	—
F14	6.22	271.06	Aspartyl-Histidine	$[M+H]^+$	$C_{10}H_{14}N_4O_5$	2.28	Histidine metabolism
F15	7.81	431.2693	Cholic acid	$[M+Na]^+$	$C_{24}H_{40}O_5$	2.08	Bile acid metabolism
F16	8.36	429.2466	3-Oxocholic acid	$[M+Na]^+$	$C_{24}H_{38}O_5$	2.46	Bile acid metabolism
F17	10.25	415.2472	Deoxycholic acid	$[M+Na]^+$	$C_{24}H_{40}O_4$	5.41	Bile acid metabolism
F18	11.02	415.278	Chenodeoxycholic acid	$[M+Na]^+$	$C_{24}H_{40}O_4$	1.93	Bile acid metabolism
F19	11.49	413.2659	Nutriacholic acid	$[M+Na]^+$	$C_{24}H_{38}O_4$	1.56	Bile acid metabolism
F20	11.5	413.2652	12-Ketodeoxycholic acid	$[M+Na]^+$	$C_{24}H_{38}O_4$	2.50	Bile acid metabolism
F21	13.25	415.2767	Allodeoxycholic acid	$[M+Na]^+$	$C_{24}H_{40}O_4$	1.56	Bile acid metabolism

Table S3. Result from ingenuity analysis with MetPA.

NO.	Pathways	Total	Expected	Hits	Raw p	-log(p)	Impact
1	Glycine, serine and threonine metabolism	32	0.48	2	8.10E-02	2.51E+00	0.03
2	Pantothenate and CoA biosynthesis	15	0.22	1	2.04E-01	1.59E+00	0.33
3	Nicotinate and nicotinamide metabolism	13	0.19	1	1.79E-01	1.72E+00	0.24
4	Primary bile acid biosynthesis	46	0.69	2	1.49E-01	1.90E+00	0.00
5	Pyruvate metabolism	22	0.33	1	2.84E-01	1.26E+00	0.10
6	Glyoxylate and dicarboxylate metabolism	16	0.24	1	2.16E-01	1.53E+00	0.04
7	Citrate cycle (TCA cycle)	20	0.30	1	2.62E-01	1.34E+00	0.03
8	Arginine and proline metabolism	44	0.66	1	4.91E-01	7.12E-01	0.02
9	Purine metabolism	68	1.02	2	2.71E-01	1.31E+00	0.00
10	Propanoate metabolism	20	0.30	2	3.45E-02	3.37E+00	0.00
11	Alanine, aspartate and glutamate metabolism	24	0.36	2	4.84E-02	3.03E+00	0.00
12	Taurine and hypotaurine metabolism	8	0.12	1	1.14E-01	2.17E+00	0.00
13	Ascorbate and aldarate metabolism	9	0.13	1	1.27E-01	2.06E+00	0.00
14	Butanoate metabolism	20	0.30	1	2.62E-01	1.34E+00	0.00
15	Cysteine and methionine metabolism	28	0.42	1	3.47E-01	1.06E+00	0.00
16	Tryptophan metabolism	41	0.61	1	4.66E-01	7.63E-01	0.00

Note: Total is the total number of compounds in the pathway; the Hits is the actually matched number from the user uploaded data; the Raw p is the original p value calculated from the enrichment analysis; the Impact and -log(p) is the pathway impact value calculated from pathway topology analysis.

Table S4. The reproducibility and precision of UPLC-Q-TOF/MS method validation under the positive ion mode using QC samples (urine samples: **A**; feces samples: **B**).

A:

NO.	RT(min)	m/z	repeatability		precision	
			$RSD(\%)_{RT}$	$RSD(\%)_{m/z}$	$RSD(\%)_{RT}$	$RSD(\%)_{m/z}$
1	0.65	144.1020	0.0000	0.0001	0.0000	0.0007
2	1.93	136.0395	0.0055	0.0003	0.0000	0.0009
3	3.84	242.1013	0.0084	0.0010	0.0000	0.0008
4	4.43	297.1447	0.0089	0.0005	0.0000	0.0013
5	5.41	178.0504	0.0071	0.0002	0.0000	0.0005
6	6.07	162.0549	0.0084	0.0001	0.0000	0.0009
7	7.72	162.0559	0.0000	0.0006	0.0832	0.0004
8	8.61	164.0708	0.0000	0.0002	0.5325	0.0110
9	9.29	255.0659	0.0000	0.0003	0.8165	0.0005
10	9.48	285.0767	0.0055	0.0005	0.0000	0.0014

B:

NO.	RT(min)	m/z	repeatability		precision	
			$RSD(\%)_{RT}$	$RSD(\%)_{m/z}$	$RSD(\%)_{RT}$	$RSD(\%)_{m/z}$
1	1.75	120.0810	0.0000	0.0006	0.0000	0.0010
2	2.34	146.0602	0.8165	0.0008	0.0000	0.0010
3	3.57	202.0893	0.5164	0.0010	0.0000	0.0004
4	5.77	595.3494	0.4083	0.0016	0.7528	0.0010
5	7.85	355.2622	0.6325	0.0026	0.6325	0.0010
6	9.87	355.2628	0.0000	0.0024	0.4083	0.0014
7	10.27	274.2736	0.5164	0.0008	0.5477	0.0010
8	10.48	318..3005	0.0000	0.0007	0.0000	0.0007
9	12.07	437.1923	0.0000	0.0015	0.0000	0.0014
10	13.26	357.2788	0.0000	0.0024	0.0000	0.0009

The RSD $(\%)_{RT}$ and RSD $(\%)_{m/z}$ values representing for the RSD of retention time and *m/z* of the selected ions based on UPLC-Q-TOF/MS method.
